# Validity and reliability of the Chinese version of the Health Literacy Scale Short-Form in the Chinese population

**DOI:** 10.1186/s12889-023-15237-2

**Published:** 2023-02-23

**Authors:** Xiaonan Sun, Ke Lv, Fei Wang, Pu Ge, Yuyao Niu, Wenli Yu, Xinying Sun, Wai-Kit Ming, Miao He, Yibo Wu

**Affiliations:** 1 Department of Social Science and Humanities, Harbin Medical University, Harbin, Heilongjiang Province China; 2 School of Public Health, China Medical University, Shenyang, Liaoning Province China; 3 State Key Laboratory of Cognition and Learning, Department of Psychology, Beijing Normal University, Beijing, China; 4 Institute of Chinese Medical Sciences-State Key Laboratory of Quality Research in Chinese Medicine, University of Macau, Macau, China; 5 Department of Public Health and Medicinal Administration, Faculty of Health Sciences, University of Macau, Macau, China; 6 Faculty of Arts and Humanities, University of Macau, Macau, China; 7 Weifang University of Science and Technology, Shouguang, Shandong Province China; 8 School of Public Health, Peking University, Beijing, China; 9 Department of Infectious Diseases and Public Health, Jockey Club College of Veterinary Medicine and Life Sciences, City University of Hong Kong, Kowloon, Hong Kong China

**Keywords:** Chinese population, Health literacy, Reliability, Validation

## Abstract

**Background:**

Health literacy is closely related to health status. Measuring public health literacy levels helps to warn of health status and manage health problems through timely interventions. The items of relevant evaluation tools are complex and numerous in China, and there is no recognized health literacy brief scale for the whole population. To translate the 12-item short-form health literacy scale (HLS-SF12) and test the validity and reliability of the Chinese version of the HLS-SF12 in the Chinese population.

**Methods:**

The HLS-SF12 was translated into Chinese using the procedures of translation, back translation, and cultural debugging. 10,951 residents were selected by quota sampling method to test the validity and reliability of the scale, and 33 people were selected to retest after 2 weeks. The reliability was tested by using internal consistency coefficient and test-retest reliability. The validity was tested by using confirmatory factor analysis, content validity, convergent validity and discriminant validity.

**Results:**

The Cronbach’s Alpha coefficient for the total scale was 0.94, and the test-retest reliability was 0.89. The Cronbach’s Alpha coefficients for the three subscales of health care, disease prevention, and health promotion respectively were 0.86, 0.86, 0.87, and the test-retest reliability respectively were 0.91, 0.79, 0.63. The confirmatory factor analysis identified a three factors model and showed nice goodness of fit indices for Chinese HLS-SF12 (GFI = 0.96, CFI = 0.97, IFI = 0.97, TLI = 0.96, and RMSEA = 0.07).

**Conclusion:**

The Chinese version of the HLS-SF12 has good reliability and validity, and can be used as a tool to evaluate the health literacy of Chinese people.

## Introduction

Health literacy is the ability of individuals to access and understand health information and to use that information to maintain and promote their own health [1]. The World Health Organization suggests that health literacy is a symbol of cognitive and social skills [2] and is an important determinant of health [3]. Limited health literacy leads to low awareness of chronic diseases, poor self-management of diseases, low utilization of health care resources, poor adherence to medication, and increased hospitalization and mortality [4–6]. This requires health literate vulnerable groups to understand and use health information in order to adequately manage their health problems. At present, the research and development of tools to measure the health literacy of public groups is active. Foreign scholars mostly use the health literacy questionnaire (HLQ), the European health literacy survey questionnaire (HLS-EU-Q) [7, 8], while scholars in China mostly use the “National Resident Health Literacy Monitoring Questionnaire” formulated by the National Health Commission [9–12].

However, these scales have too much content, especially the “National Residents Health Literacy Monitoring Questionnaire” has 80 items, which generally take a long time to measure, often affect the enthusiasm of the assessment population, and limit the large-scale application to a certain extent [13]. At the same time, “National Resident Health Literacy Monitoring Questionnaire” limits the applicable population of the questionnaire with a longer number of entries, and basically scholars limit the applicable age of the questionnaire to under 70 years old.

The 12-item short-form health literacy scale (HLS-SF12) is developed by Tuyen V. Duong for people over the age of 15 in Asia, which is on the basis of the European health literacy survey questionnaire-47(HLS-EU-Q47). The HLS-F12 as an assessment tool retains the original conceptual framework of HLS-EU-Q47 and is an optimized version for fast, comprehensive and effective measurement of health literacy [14]. A short assessment tool can be incorporated into assessment questionnaires such as patient visit assessments to quickly screen out vulnerable groups in health literacy and facilitate the implementation of targeted health education and assessment of intervention effects [7, 15]. The HLS-SF12 scale has been applied by foreign scholars in studies of patients in general outpatient clinics, orthopedic and Chinese medicine departments [16], as well as validated and analyzed the factors associated with health literacy in this group among rural residents in Vietnam [17], and explored the relationship between health literacy and anxiety and depression among health workers and outpatients during the New Crown pandemic, which has been useful in developing mental health and health quality by providing evidence to support government and organizational strategies to improve mental health and health quality [18, 19]. Several studies have shown that the scale has good reliability and validity, and is generalizable in across-cultural backgrounds, geographic differences and social group differences, and can be used as a valid measurement tool for applying health literacy to multiple groups.

At present, there is no recognized health literacy scale short-form for the whole population in China, and the relevant health literacy measurement questionnaires have many items and are complicated, and there is a lack of relevant studies on whether the HLS-SF12 can be used in the Chinese population. Therefore, this study introduces the HLS-SF12, evaluates the reliability and validity in Chinese population, and forms a Chinese version of the health literacy scale short-form to provide a rapid and effective measurement tool for the health literacy study of the whole population in China.

## Objects and Methods

### Sample

#### Pre-test samples

Taking the poor cognitive ability of minors and older people into account, the researchers used quota sampling according to age, gender, and urban-rural distribution, and distributed questionnaires online in June 2021. Eventually there were 20 adolescents aged 12–18 years and 25 elders were selected for the pre-test.

#### Large samples of formal research

Conducting this survey from July 10, 2021 to September 15, 2021, a multi-stage sampling method was used to directly include the provincial capitals of 23 Chinese provinces and 5 autonomous regions, 4 municipalities directly under the Central Government (Beijing, Tianjin, Shanghai, Chongqing), and 2–6 cities in each of the non-capital prefecture-level administrative regions of each province and autonomous region using the random number table method, for a total of 120 cities. At least one surveyor or one survey team was recruited from each city. The enumerators were required to make the gender, age, and urban-rural distribution of the obtained sample basically match the demographic characteristics based on the results of the “7th National Population Census in 2021”. Inclusion criteria were: ① age ≥ 12 years; ② nationality of the People’s Republic of China; ③ permanent residence in China (time spent away from home ≤ 1 month per year); ④ voluntary participation in the study and completion of the informed consent form. Exclusion criteria: ① those who were delirious or mentally abnormal; ② those who were participating in other similar research subjects; ③ those who were cognitive impairment and dyslexia. The questionnaires were collected and then logically checked and data screened by two people back to back. This study was ethically reviewed (JNUKY-2021-018).

A total of 11,668 residents were surveyed and 10,951 valid questionnaires were returned, with an effective rate of 93.85%. A total of 10,951 cases of residents aged 12 years and above were included for the reliability test of the scale. See 3.2 General information on the sample for details.

#### The sample of the test-retest

Using the random number table method, the researchers randomly invited 39 respondents in the formal survey to retest after 2 weeks. After receiving responses from respondents and conducting logic checks, 33 respondents eventually participated in the test-retest reliability survey.

#### Sample size calculation

In order to ensure the validity of the analysis of pretest samples and test-retest reliability samples, the researchers usually select 30 or more samples as much as possible [20, 21]. In this study, a total of 45 pretest respondents and 33 test-retest reliability respondents were selected, which met the sample size requirements of pretest and test-retest reliability.

In this study, the minimum sample size required for formal research was calculated [22]: 223 participants were required to achieve a statistical power of 95% by assuming an intraclass correlation coefficient (ICC) of 0.90 and a Type I error probability α of 0.05. A total of 10,951 valid data were collected in the formal study in this study, indicating that the sample size was sufficient for subsequent data analysis.

### Research tools

#### General information questionnaire

It was compiled by the researchers himself and included the survey respondents’ gender, age, ethnicity, type of household registration, place of residence, political affiliation, highest education, marital status, birth status, residence status, mode of bearing medical expenses, and per capita monthly household income.

#### HLS-SF12

The HLS-SF12, developed by Tuyen V. Duong et al. and applicable to public health literacy measurement, includes 3 dimensions of health care, disease prevention and health promotion, with 12 entries, each rated on a 4-point scale (1 = very difficult, 2 = difficult, 3 = easy, 4 = very easy), using a formula to calculate a standardized HL index ranging from 0 to 50, with higher indices representing higher level of health literacy. The formula is, index = (mean − 1) * (50/3), where the mean is the average of all items involved for each individual, 1 is the minimum possible value of the mean (when the minimum value of the index is 0), 3 is the range of maximum value (4) minus minimum value (1) of the average score of the respondent for each question, 3 = 4 − 1, and 50 is the maximum value of the index. Tuyen V. Duong reports the Cronbach’ s Alpha of the Health Literacy Scale Short Form was > 0.70, the Cronbach’s Alpha coefficients of 0.49 to 0.72 for the Health Care sub scale, 0.64 to 0.77 for the Disease Prevention sub scale, and 0.64 to 0.77 for the Health Promotion sub scale, and the Cronbach ‘s Alpha coefficients ranged from 0.59 to 0.81, indicating that the scales had good internal consistency. The Chinese version of the HLS-SF12 was used for the test after the transcultural adaptation.

### Research Methodology

In this study, after contacting the original scale developer, Tuyen V. Duong, by email to obtain authorization for the use and translation revision of the scale, the standard procedures for scale Chinesization, such as translation, back translation, and cultural debugging, were used to Chinesize the Health Literacy Short Form [23] .

#### Translation and back-translation of the scale

Two native Chinese and fluent in English masters (one medical and one English) translated the scale independently, and then one native Chinese and fluent in English master compared and analyzed the first two translated versions, and discussed with the first two translators to form a composite draft of the Chinese version of the scale. Afterwards, two English translators with no medical background back-translated the composite version of the Chinese version of the scale separately without knowing the content of the scale. Finally, a medical master who was a native Chinese speaker and fluent in English and did not participate in the translation and back-translation process compared the back-translated version of the scale with the original scale and revised it together with the two back-translators to form the first draft of the Chinese version of the health literacy scale.

#### Cross-cultural debugging of the scale

An expert group consisting of five experts in the fields of humanistic medicine, social medicine, health statistics, health career management and medical English and all translators (including forward translators, translation synthesizers and back translators) reviewed and debugged the entries of the first draft of the Chinese version of the scale from four aspects: semantics, idiom, experience and equivalence of concepts, according to the actual situation and language expression habits in China.

Before the formal survey, the researchers selected 20 adolescents aged 12–18 years and 25 elders for the pre-test using a paper-based general information questionnaire and a Chinese version of the health literacy scale short form. Firstly, the researchers explained the purpose and significance of the study to the participants and obtained informed consent. After the participants completed the scale, the researchers asked about the linguistic appropriateness, semantic comprehension and content acceptability of each item.

#### Statistical analysis

SPSS 26.0 and AMOS 26.0 were used for the data entry and analysis. Count data were described as frequencies and percentages, item analysis was performed by correlation coefficient, extreme group method, CITC method, scale reliability was evaluated by internal consistency Cronbach’s alpha coefficient, test-retest reliability and fold half coefficient; scale validity was analyzed by content validity, structural validity (validation factor analysis), convergent validity and discriminant validity. All data were tested using a two-sided test, and *p* < 0.05 indicated that the differences were statistically significant.

## Results

### The result of sinicization

The HLS-SF12 was translated into Chinese using the procedures of translation, back translation, and cultural debugging. On the basis of respecting the original meaning of the scale, the researchers modified the expressions that did not conform to the Chinese mainland language habits, such as changing “understand the leaflets that come with your medicine” to “understand the instructions that come with your medicine”, and changing “judge which everyday behavior is related to your health” to “determine which daily behaviors can have an impact on your health” etc. All 12 entries were eventually retained, resulting in a Chinese version of the health literacy short form (see Appendix) .

### General information on the sample

Among them, 4994 cases (45.6%) were male and 5957 cases (54.4%) were female; 1008 cases (9.2%) were aged 12–18, 3600 cases (32.9%) were aged 19–30, 1732 cases (15.8%) were aged 31–40, 2481 cases (22.7%) were aged 41–50, 1006 cases (9.2%) were aged 51–59, and 1124 cases (10.2%) were aged 60 years or older; married 6219 cases (56.8%); urban residents 7962 cases (72.7%), rural residents 2989 cases (27.3%); non-agricultural households 6326 cases (57.8%), agricultural households 4625 cases (42.2%); college education and above 6456 cases (59.0%); medical expenses were borne by Resident medical insurance 5294 cases (48.3%) (see Table 1).

**Table 1:** General demographic characteristics of survey participants

Characteristic	Number (%)		Characteristic	Number (%)	
sex			Permanent residence		
male	4994	(45.6)	Cities and towns	7962	(72.7)
women	5957	(54.4)	Rural	2989	(27.3)
Age range (years)			Nationality		
12 ~ 18	1008	(9.2)	Han ethnic group	10,310	(94.1)
19 ~ 30	3600	(32.9)	National minority	641	(5.9)
31 ~ 40	1732	(15.8)	Marital status		
41 ~ 50	2481	(22.7)	Unmarried	4291	(39.2)
51 ~ 59	1006	(9.2)	Married	6219	(56.8)
60–70	508	(4.6)	Divorced or widowed	441	(4.0)
71 ~ 80	512	(4.7)	Professional status		
≥ 81	104	(0.9)	Students	3236	(29.5)
Highest level of education			Be in post	4637	(42.3)
No formal academic education	375	(3.4)	Retirement	883	(8.1)
Secondary school	744	(6.8)	No fixed occupation	2195	(20.0)
Junior high school	1421	(13.0)	Method of covering medical expenses		
Secondary or high school	1955	(17.8)	Resident health insurance	5294	(48.3)
Tertiary and above	6456	(59.0)	Employee health insurance	2935	(26.8)
Nature of household			Publicly funded, self-funded and commercial health insurance	2722	(24.9)
Non-agricultural	6326	(57.8)	Monthly per capita household income(¥)		
Agriculture	4625	(42.2)	≤ 1500	1056	(9.6)
Region			1501–3000	2168	(19.8)
Eastern part	5667	(51.7)	3001–4500	2323	(21.2)
Middle part	2969	(27.1)	4501–6000	1903	(17.4)
Western part	2315	(21.1)	≥ 6001	3501	(32.0)
Status of receipt of grants			Religious affiliation or non-affiliation		
Government grants	1339	(12.2)	Be	319	(2.9)
Social grants	249	(2.3)	Deny	10,632	(97.1)
No grant received	6459	(59.0)	Alcohol consumption		
Unclear	2904	(26.5)	Drank, within the last 30 days	3130	(28.6)
Number of drugs currently being taken			Drank, 30 days ago.	1299	(11.9)
Not taking any medication	8926	(81.5)	Never had one.	6522	(59.6)
1 type	924	(8.4)	Smoking status		
2 kinds	609	(5.6)	Cigarette smoking	1395	(12.7)
≥ 3 types	492	(4.5)	Quit smoking	783	(7.2)
			Never smoked	8773	(80.1)

### HL index scores

The statistical results of the scale scores are shown in Table 2. The range of HL index scores for the HLS-SF12 total scale is 0 to 50 with a mean of (34.31 ± 8.40). The percentage of lowest scorers on the HLS-SF12 total scale and subscales ranges 0.59 ~ 0.88% and the percentage of highest scorers ranges from 9.11 ~ 14.66%, both below 15%, indicating no ceiling or floor effects exist [24].

**Table 2:** The HL index scores situation (n = 10,951)

Dimensionality	Minimum value	Maximum value	x ± s	Floor effect %	Ceiling effect %
Health care	0	50	33.89 ± 9.34	0.88	13.13
Disease prevention	0	50	34.34 ± 8.93	0.79	13.21
Health promotion	0	50	34.70 ± 8.98	0.75	14.66
Summary table	0	50	34.31 ± 8.40	0.59	9.11

### Item analysis

Using the critical proportion method, the total score of each sample questionnaire was calculated, ranked by the size of the total score, and the top 27% of the ranked total score was calculated as an indicator for determining the high and low subgroups, and an independent samples t-test was conducted on the total scores of the high and low subgroups, and the results showed that there was a significant difference between the scores of the high and low subgroups on all entries (*t=-138.36, p < 0.001*). Pearson correlation was used to test the correlation between the scores of each entry and the total score, and the results showed that there was a significant and high correlation between the scores of the entries of the health care dimension, disease prevention dimension, and health promotion dimension of the health literacy scale short form and the total score, with correlation coefficients ranging from 0.75 to 0.81, 0.77 to 0.81, and 0.93 to 0.82. The overall correlation coefficients of the items, CITC, were all above 0.40, and combined with the Cronbach’s alpha coefficients after deletion it is clear that the internal consistency coefficients did not change much after deletion of the items (see Table 3). The results of item analysis indicate that the Chinese version of HLS-SF12 has good discriminatory power.

**Table 3:** Item-total correlation coefficients of the Chinese version of HLS-SF12

Scale question items	After the deletion of the item Scale Mean	After the deletion of the item standard deviation	Amended item Relevance to total	Cronbach’s Alpha after removal of terms
A1	33.70	30.82	0.70	0.94
A2	33.63	30.55	0.76	0.93
A3	33.80	30.51	0.71	0.94
A4	33.55	31.18	0.70	0.94
B1	33.75	30.82	0.72	0.94
B2	33.58	30.97	0.75	0.94
B3	33.66	30.59	0.75	0.94
B4	33.58	31.06	0.77	0.93
C1	33.62	31.04	0.74	0.94
C2	33.61	30.67	0.77	0.93
C3	33.55	31.31	0.74	0.94
C4	33.71	30.91	0.67	0.94

### Reliability analysis of the Chinese version of the health literacy scale short form

The Cronbach’s alpha coefficient for the Chinese version of the health literacy scale short form was 0.94, the Spearman-Brown coefficient was 0.93, and the split-half reliability of the scale was 0.91. The Cronbach’s alpha coefficient for the health care subscale was 0.86, the Cronbach’s alpha coefficient for the disease prevention subscale was 0.86, and the Cronbach’s alpha coefficient for the health promotion subscale was 0.87. In order to test the stability of the scale, the researchers included 33 participants for the test-retest reliability survey, and the results showed that the test-retest reliability of the Chinese version of the HLS-SF12 after two weeks was 0.89. The test-retest reliability of the health care subscale was 0.91, the test-retest reliability of the disease prevention subscale was 0.79, and the test-retest reliability of the health promotion subscale was 0.63.

### Validity analysis of the Chinese version of the health literacy scale short form

#### Construct validity - validation factor analysis

**Fig. 1:**
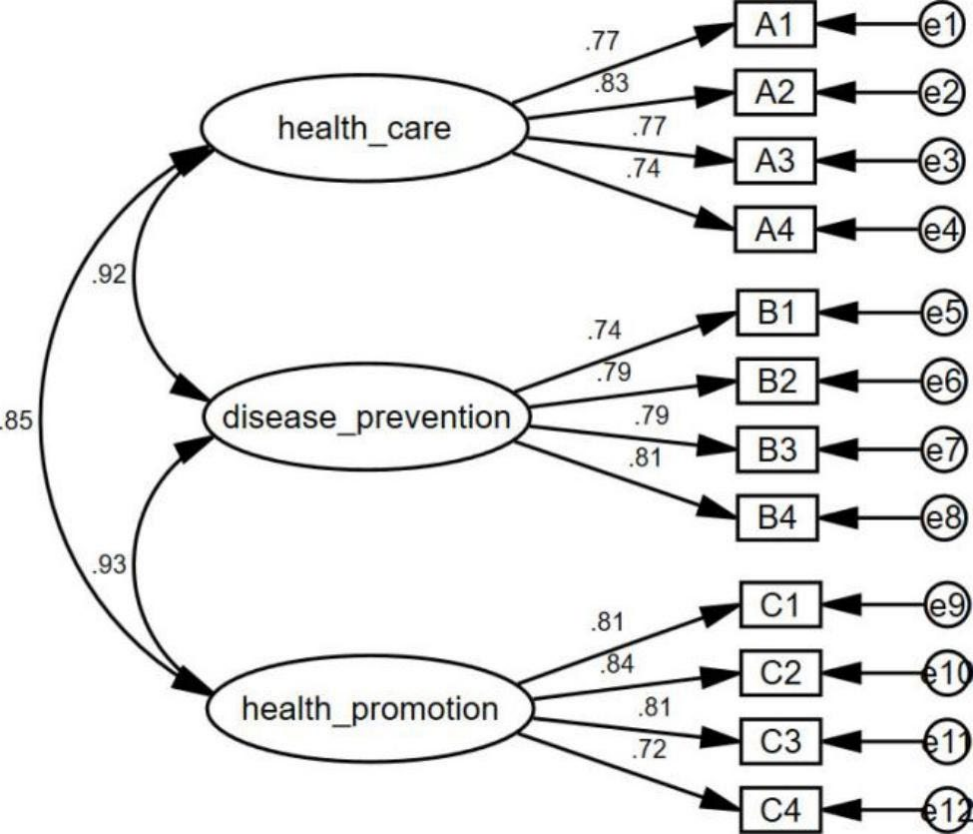
The confirmatory factor model of the Chinese version of HLS-SF12

The validation factor analysis was used to test the structural validity of the scale, and the scale was validated according to the one-factor structural model of the original scale. The Root Mean Square Error of Approximation (RMSEA) of the model is 0.07; The Goodness of Fit Index (GFI) of the model is 0.96; The Comparative Fit Index (CFI) of the mode is 0.97; The Incremental Fit Index (IFI) of the model is 0.97; The Tucker-Lewis Index (TLI) of the model is 0.96, all values are greater than 0.90, and the results fit well. Collectively, it appears that the models for health care, disease prevention, and health promotion fit well, and the results of the validated factor analysis are shown in Fig. 1.

#### Content validity

The expert consultation method was used to ensure the content validity of the Chinese version of the HLS-SF12. Experts from multiple fields (including social medicine, health statistics, health care management, psychology, humanistic medicine, clinical medicine, etc.) were invited for the expert consultation and discussion on June 7, June 11, June 15, June 18, July 3, and July 8, 2021. The experts consulted were all senior in rank and regionally representative. The content validity index questionnaire was scored on a 4-point scale (1 = Unrelated, 2 = Weak related, 3 = Stronger related, 4 = Strongest related) to assess the appropriateness of the items. The item level content validity index (I-CVI) and the scale-level content validity index (S-CVI) were calculated separately for each item of the scale and the total scale, and the results showed that the I-CVI of all 12 items in the scale was greater than 0.78 and the K* was greater than 0.74, suggesting that the content validity of the items was excellent. The number of entries rated 3 or 4 by all experts in the scale was 3, so the S-CVI/UA was 0.25, which was lower than 0.80. Calculating the mean for each I-CVI yielded an S-CVI/Ave of 0.95, which was greater than 0.90. Overall, the scale has good content validity and can be used as a valid evaluation tool.

#### Convergent validity

The results of convergent validity are shown in Table 4. it can be seen that the Average Variance Extracted (AVE) values of the three dimensions of health care, disease prevention and health promotion are greater than 0.50 and the Composite Reliability (CR) values of each dimension are greater than 0.70, indicating high convergent validity.

**Table 4:** Factor loading of the Chinese version of HLS-SF12

Dimensionality	Item	Factor load	AVE	CR
Health care	A1	0.77	0.61	0.86
	A2	0.83		
	A3	0.77		
	A4	0.74		
Disease prevention	B1	0.74	0.62	0.87
	B2	0.79		
	B3	0.79		
	B4	0.81		
Health promotion	C1	0.81	0.63	0.87
	C2	0.84		
	C3	0.81		
	C4	0.72		

#### Discriminant validity

The discriminant test was performed using the chi-square difference test for two-by-two comparison between the constructs, and the significance of the difference between the chi-square values of the unrestricted and restricted models using the structural equation approach. The results of the discriminant validity are shown in Table 5. The other models were worse for each fit indicator compared to the original model and passed the chi-square test at a significance level of 0.001, indicating good model discriminant validity.

**Table 5:** Discrimination validity of the Chinese version of HLS-SF12

Number	Models	*χ2*	*df*	*χ2/df*	RMSEA	GFI	CFI	Model Comparison	*Δχ2*	*Δdf*
1	Original model	2702.70	51	52.99	0.07	0.96	0.97			
2	Two-factor model I	3827.97	53	72.23	0.08	0.94	0.96	2 vs. 1	1125.27***	2
3	Two-factor model II	3886.26	53	73.33	0.08	0.94	0.96	3 vs. 1	1183.56***	2
4	One-factor model	5618.57	54	104.05	0.10	0.91	0.94	4 vs. 1	2915.87***	3
**Note**: ***P < 0.001.										
Two-factor model I: F1, F2 + F3										
Two-factor model II: F1 + F2, F3										
One-factor model: F1, F2, F3										

## Discussion

The HLS-SF12 was developed based on the HLS-EU-Q47. The HLS-SF12 retains the original 12 items and presents the original architecture of the HLS-EU-Q47 with good reliability [14]. This will facilitate a simple and accurate assessment of health literacy in a larger Asian population or clinical setting. In contrast, the HLS-SF12 scale has not yet been translated into the Simplified Chinese Characters, and its applicability to the whole population in China is not clear. Therefore, this study was conducted to investigate the application of the Chinese version of the HLS-SF12 in the Chinese population after a rigorous translation and reliability measurement of the scale.

This study adapts the source scale cross-culturally from four aspects: semantic equivalence, idiomatic equivalence, empirical equivalence and conceptual equivalence, so that the language expression is more suitable for the language habits and social reality of the Chinese mainland. In order to make the respondents’ understanding smoother, in the process of sinicization, we replaced “the leaflets that come with your medicine?” in the original text of the scale with “the instructions of the drug”. At the same time, according to the daily exercise of the Chinese mainland people, “the sports club or exercise class” is modified to “sports organization or fitness classes”.

The authors of the original scale developed and verified the Traditional Chinese Characters version of HLS-SF12 in Taiwan. However, there are differences in the cultural backgrounds of Taiwan and the Chinese mainland. And across the strait use different fonts, with Taiwan using Traditional Chinese Characters while the Chinese mainland using Simplified Chinese Characters. In the Traditional Chinese Characters version of the HLS-SF12, item 10 is “瞭解媒體(電視、網站或其它媒體)在促進健康議題上提供的資訊嗎” (to understand the information provided by the media on health promotion issues), and the words “資訊” and “議題” are not the most appropriate in the Chinese mainland idioms. Therefore, on the basis of respecting the original English scale, we revised this entry to “理解媒体上有关如何变得更健康的信息” (understand the information in the media about how to become healthier). In addition, in the Traditional Chinese Characters version of the scale, “那些” (those) is used to represent the interrogative tone (e.g能判斷你需要接受的是那種疫苗),while in the Chinese mainland idiom, we usually use “哪些” (which) to express it.

Item analysis was performed using the extreme group method, the correlation coefficient method and the CITC method, and the results showed that the entries in the Chinese version of the HLS-SF12 had good discrimination, as well as a high correlation with the total score of the scale, so all 12 items of the short form were retained.

The reliability test used Cronbach’s alpha coefficient and test-retest reliability to evaluate the internal consistency and stability of the scale. The Cronbach’s alpha coefficient of the Chinese version of the HLS-SF12 was 0.94, the split-half reliability was 0.91, and the Cronbach ‘s Alpha coefficients were all greater than 0.80, indicating that the internal consistency of the scale was good. The test-retest reliability mainly examined the stability of the scale across time, and if both measurements were greater than 0.70, it was considered to have good test-retest reliability [25]. In this study, 33 respondents were selected and retested after 2 weeks, and their test-retest reliability was 0.89, which has good stability across time.

Validity tests were conducted using structural validity, content validity, convergent validity and discriminant validity, and RMSEA ≤ 0.08, GFI > 0.90, CFI > 0.90, IFI > 0.90, TLI > 0.90 were chosen as the acceptance criteria, where the *χ2/df* reliability was not good in the case of large samples, so the model fitted well, the structural validity was good, and the expert consultation method guaranteed good content validity, while the results show that the scale has good convergent and discriminant validity [26].

The Chinese version of the HLS-SF12 in this study has only 12 entries, is clearly expressed, and is more in line with the cognitive level of the adolescent and elderly population than the commonly used health literacy monitoring questionnaires in China, and is simple and easy to administer. Both the experts consulted in the relevant fields and the pre-tested research subjects thought that the questionnaire was clearly expressed and easy to understand, so the applicability of the original scale could be extended from people over 15 years of age to people over 12 years of age. Meanwhile, supported by Binh N. Do et al. [27] study exploring health literacy among 60–85 year elders, the reliability test of this study chose to expand the population of health literacy measurement from 60 to 69 years old in the current Chinese study to a higher age group, which is in line with the national situation of population aging in China. The scale can provide a simpler and more effective measurement tool for the investigation of the current situation of health literacy and the analysis of the influencing factors in China. It is important to note some limitations of this cross-sectional study. First, it is difficult for us to achieve complete randomization in the selection of study subjects, and sample selection bias is inevitable. However, we used large samples of data across the country, and the results were relatively reliable. Second, the current health literacy assessment tools include not only the HLS-SF12, but also the HLQ, HLS-EU-Q47 and other scales, which should be combined with the above scales as validity scales in future studies to further validate the reliability of the scale, so as to facilitate cross-sectional comparative studies among multiple scales. Third, our health literacy assessments are subjective reports by respondents based on their own perceptions, which can lead to reporting bias. In future studies. We propose to add objective measures such as respondents’ physical condition to aid the research.

## Conclusion

This study introduced the HLS-SF12 from abroad and measured the applicability of the Chinese version of the HLS-SF12 in our population, and the results showed that the Chinese version of the HLS-SF12 has good reliability and validity. The scale has 12 items, and each item is clearly expressed and easily understood by the adolescent and elderly populations, which can provide a tool for assessing the current situation and influencing factors of health literacy in China.

## Data Availability

The datasets used and/or analysed during the current study are available from the corresponding author on reasonable request.

## List of abbreviations

*HLS-SF12*: 12-item short-form health literacy scale
*HLQ*: health literacy questionnaire
*HLS-EU-Q*: European health literacy survey questionnaire
*HLS-EU-Q47*: European health literacy survey questionnaire-47
*RMSEA*: Root Mean Square Error of Approximation
*GFI*: Goodness of Fit Index
*CFI*: Comparative Fit Index
*IFI*: Incremental Fit Index
*TLI*: Tucker-Lewis Index
*I-CVI*: item level content validity index
*S-CVI*: scale-level content validity index
*AVE*: Average Variance Extracted
*CR*: Composite Reliability

## References

[1] Ad Hoc Committee on Health Literacy for the Council on Scientific Affairs, American Medical Association (1999). Health literacy: report of the Council on Health literacy: report of the Council on Scientific Affairs[J]. JAMA.

[2] Bu Ru’e (2012). Research on the health literacy evaluation index system based on public health emergencies [D].

[3] Sheiham A, Commission on Social Determinants of Health (CSDH) (2009). Closing the gap in a generation: Health Equity through Action on Social Determinants of Health[J]. Community Dent Health.

[4] Muir KW, Santiago-Turla C, Stinnett SS (2006). Health literacy and adherence to Glaucoma Therapy[J]. Am J Ophthalmol.

[5] Baker DW, Gazmararian JA, Williams MV (2002). Functional health literacy and the risk of hospital admission among Medicare managed care enrollees[J]. Am J Public Health.

[6] Sudore RL, Yaffe K, Satterfield S (2006). Limited literacy and mortality in the elderly[J]. J Gen Intern Med.

[7] Li T, Yi Qiaoyun,Sun Mei. Research progress of health literacy assessment tools[J].Journal of Liberation Army Nursing,2015,32(18):29–32.

[8] Ouyang Yu,Wang Xiuhua,Yang Chen,Tan Zheyu. Research progress on health literacy assessment tools for older adults[J].Journal of Liberation Army Nursing,2018,35(02):39–43 + 48.

[9] Zhou L, Ma L, Luo Y, Xu JD. Survey on the current situation of health literacy among rural residents in Hubei Province and analysis of influencing factors[J].China Health Education,2019,35(08):697–700 + 715.

[10] Jin Fei Fei. Development and evaluation of health literacy scale for rural residents in China[D]. Beijing:China Center for Disease Control and Prevention; 2018.

[11] Huang Weidong S, Pinghui (2010). Analysis of factors affecting health literacy among the elderly[J]. Chin J Gerontol.

[12] Qian Xiaobo L, Jinghua Z, Xiumin (2013). A comparison of health literacy-related issues among urban and rural residents in Jilin Province[J]. Chin J Gerontol.

[13] Al Sayah F, Williams B, Johnson JA (2013). Measuring health literacy in individuals with diabetes: a systematic review and evaluation of available measures[J]. Health Educ Behav.

[14] Duong TV, Aringazina A, Kayupova G (2019). Development and validation of a new short-form health literacy instrument (HLS-SF12) for the General Public in six asian Countries[J]. HLRP Health Literacy Research and Practice.

[15] Cawthon C, Mion LC, Willens DE (2014). Implementing Routine Health literacy Assessment in Hospital and Primary Care Patients[J]. Joint Comm J Qual Patient Saf.

[16] Duong TV, Chang PW, Yang SH (2017). A New Comprehensive short-form health literacy Survey Tool for patients in General[J]. Asian Nurs Res (Korean Soc Nurs Sci).

[17] Duong TV, Nguyen T, Pham KM (2019). Validation of the short-form health literacy questionnaire (HLS-SF12) and its determinants among people living in rural Areas in Vietnam[J]. Int J Environ Res Public Health.

[18] Tran TV, Nguyen HC, Pham LV (2020). Impacts and interactions of COVID-19 response involvement, health-related behaviours, health literacy on anxiety depression and health-related quality of life among healthcare workers: a cross-sectional study [J]. BMJ Open.

[19] Pham KM, Pham LV, Phan DT et al. Healthy Dietary Intake Behavior Potentially Modifies the Negative Effect of COVID-19 Lockdown on Depression: a Hospital and Health Center Survey [J]. Front Nutr, 2020, 7: 581043.2020, 7:581043. 10.3389/fnut.2020.581043. 10.3389/fnut.2020.581043 PMC7701254 33304917

[20] Perneger TV, Courvoisier DS, Hudelson PM, Gayet-Ageron A. Sample size for pre-tests of questionnaires[J]. Qual Life Res.2015;24(1):147–151. 10.1007/s11136-014-0752-2. 10.1007/s11136-014-0752-2 25008261

[21] Wang Fei, Tang Jingqi, Sun Xiaonan, et al. Design and development of scales within the primary care domain: practical steps and statistical methods[J]. Chinese Gen Pract. 2022. [Epub ahead of print]. 10.12114/j.issn.1007-9572.2022.0819.

[22] Temel G, Erdogan S (2017). Determining the sample size in agreement studies[J]. Marmara Med J.

[23] Jones PS, Lee JW, Phillips LR (2001). An adaptation of Brislin’s translation model for cross-cultural research. Nurs Res.

[24] Terwee CB (2007). Quality criteria were proposed for measurement properties of health status questionnaires.[J]. J Clin Epidemiol vol.

[25] Wu ML. SPSS statistical applications in practice [M].Beijing:China Railway Publishing House, 2000:7–54.

[26] Wu ML (2010). Statistical analysis of questionnaires in practice: SPSS operations and applications [M].

[27] Do BN, Nguyen PA, Pham KM, et al. Determinants of health literacy and its Associations with Health-Related behaviors, Depression among the older people with and without suspected COVID-19 symptoms: a multi-institutional study [J]. Front Public Health. 2020;8. 10.3389/fpubh.2020.581746. 10.3389/fpubh.2020.581746 PMC7703185 33313037

